# Association between brain metastasis from lung cancer and the serum level of myelin basic protein

**DOI:** 10.3892/etm.2015.2195

**Published:** 2015-01-20

**Authors:** WEI LIU, JING ZHAO, YUJUAN WEI

**Affiliations:** Department of Respiratory Medicine, The People’s Hospital of Rizhao, Rizhao, Shandong 276826, P.R. China

**Keywords:** myelin basic protein, brain metastasis, lung cancer

## Abstract

The aim of the present study was to determine the association between the expression of myelin basic protein in the serum and the metastasis of lung cancer to the brain. A total of 68 lung cancer patients, treated in the Department of Respiratory Medicine of the People’s Hospital of Rizhao (Rizhao, China), were divided into two groups, those with brain metastasis (32 cases) and those without brain metastasis (36 cases). The expression levels of myelin basic protein were measured for all the patients. The results indicated that the expression levels of myelin basic protein in the brain metastasis group were significantly higher when compared with those in the group without metastasis (P<0.05). However, there was no statistically significant correlation between the size of the brain metastasis and the expression levels of myelin basic protein (P>0.05). Furthermore, no statistically significant difference was found in the average level of myelin basic protein between the two subgroups of patients with brain tumor diameters of >1.5 cm and <1.5 cm (P>0.05). Therefore, the results demonstrated a statistically significant correlation between the expression of myelin basic protein in the serum and the metastasis of lung cancer to the brain. Myelin basic protein may thus prove useful in the early diagnosis of brain metastases in lung cancer patients.

## Introduction

It has been reported that >150,000 patients with cancer are diagnosed with brain metastasis each year, with lung cancer being the most common origin site for the metastasis (1). The outcome of patients with brain metastases from lung cancer is poor, with a median survival time of only one or two months for untreated patients and six months for patients who undergo surgery, radiotherapy or chemotherapy (2,3). Early diagnosis and treatment of brain metastasis may improve the therapeutic outcomes and quality of life of lung cancer patients. The diagnosis of brain metastasis depends primarily on imaging (4). However, in certain cases, particularly for small metastases in the brain, imaging examination is limited, which may result in a missed diagnosis. Therefore, finding a feasible method for the early detection of the metastasis of lung cancer to the brain is critical.

Myelin basic protein is a major structural component of myelin sheaths in the central nervous system, functioning to stabilize the structure and function of myelin sheaths (5). When the central nervous system is damaged, the function of the blood-brain barrier is affected and the concentration of myelin basic protein in the serum may increase notably. The majority of studies have indicated that myelin basic protein functions as a sensitive marker for injury of the blood-brain barrier (6–8). The expression levels of myelin basic protein are widely considered to be closely correlated with the severity of the brain trauma; however, not all studies have supported this view (9). The more severe the brain trauma, the more damage may occur to the blood-brain barrier, leading to higher levels of myelin basic protein in the serum. Berger *et al* (10) found that the expression of myelin basic protein increased following brain trauma and that the increase in myelin basic protein levels correlated closely with the category and extent of brain trauma. Levels of myelin basic protein exhibited a larger increase in cases of diffuse axonal injury, intracerebral hematoma and subdural hematoma when compared with cases of epidural hematoma (10). In addition, Baker *et al* used the concentration of myelin basic protein in the serum to evaluate the treatment outcome of brain injuries (11).

In cases of brain metastasis from lung cancer, the development of a metastatic tumor may damage the blood-brain barrier. Therefore, in the present study, the concentration of myelin basic protein was hypothesized to increase in the serum of lung cancer patients with a brain metastasis. If verified, this finding may prove helpful for physicians in the early diagnosis of the spread of lung cancer to the brain. However, to date, no studies have been published on this topic in the English literature.

In the current study, the expression levels of myelin basic protein were analyzed in 68 lung cancer patients with or without brain metastasis, treated at the Department of Respiratory Medicine of the People’s Hospital of Rizhao (Rizhao, China). The aim of the study was to determine the association between the expression of myelin basic protein and brain metastasis of lung cancer, in order to help physicians establish an earlier diagnosis.

## Materials and methods

### Patients

A total of 68 patients with lung cancer were recruited from the Department of Respiratory Medicine of the People’s Hospital of Rizhao between May 2011 and December 2013. Among the 68 lung cancer cases, 32 patients exhibited brain metastasis and 36 patients did not. The diagnosis of brain metastasis and lung cancer was based on computed tomography (CT) or magnetic resonance imaging (MRI) examinations. The patients in the brain metastasis group were further divided into two subgroups, according to the diameter of the metastatic tumor; the subgroup with a tumor diameter of ≤1.5 cm (n=10) and the subgroup with a tumor diameter of >1.5 cm (n=22). In order to facilitate the study, only patients with a single metastatic brain tumor were included. All participants provided written informed consent prior to the collection of blood samples, and the study was approved by the Ethics Committee of the People’s Hospital of Rizhao.

### Measurement of myelin basic protein

A 5-ml fasting venous blood sample was collected from each patient and stored in a test tube. The samples were centrifuged at 855 × g for 30 min, and the serum was separated and stored in a refrigerator at −70°C for subsequent analysis. The expression levels of myelin basic protein were measured using an enzyme-linked immunosorbent assay method with a human myelin basic protein ELISA kit (Fenyu Biotech, Shenzhen, China).

### Statistical analysis

Statistical analysis was performed using SPSS 17.0 software (SPSS, Inc., Chicago, IL, USA). The expression levels of myelin basic protein in the serum were compared between groups using the independent two-sample t-test. Comparisons of lung cancer category and gender between the two groups were carried out using the χ^2^ test, and the comparison of age between the two groups was performed using analysis of variance. Spearman’s correlation analysis was performed to assess the correlation between the size of the brain metastasis and the expression levels of myelin basic protein in the brain metastasis group. P<0.05 was considered to indicate a statistically significant difference.

## Results

### Patient clinical data

In total, 68 lung cancer cases were included and divided into a group with brain metastasis and a group without brain metastasis. The basic clinical data of the two groups are listed in Table I. There were no statistically significant differences in age, gender or lung cancer category between the two groups (P>0.05).

### Levels of myelin basic protein increase in brain metastasis patients

Average levels of myelin basic protein were 4.51 μg/l (range, 3.75–5.97 μg/l) and 1.38 μg/l (range, 0.69–2.54 μg/l) in the groups with and without brain metastasis, respectively. The level of myelin basic protein in the group with brain metastasis was significantly higher when compared with the group without brain metastasis (P<0.05).

### Correlation between the size of the metastatic brain tumor and the levels of myelin basic protein

**Figure 1:**
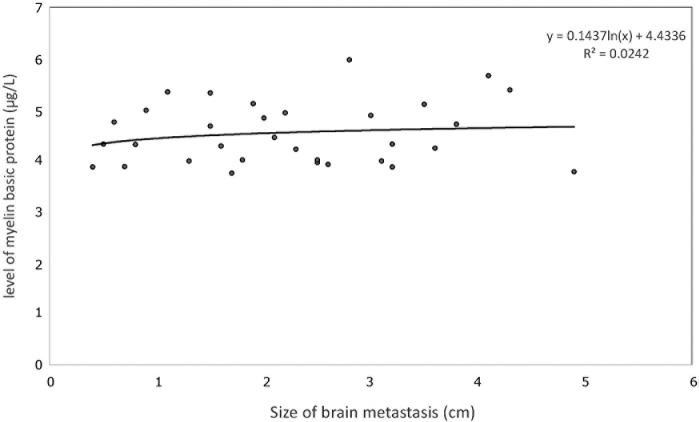
Correlation between the level of myelin basic protein and the size of the brain metastasis.

In the lung cancer with brain metastasis group, the correlation between the size of brain metastasis and the level of myelin basic protein was analyzed (Fig. 1), and the R^2^ value was determined to be 0.0242. Thus, there was no significant correlation between the size of the brain metastasis and the level of myelin basic protein (P>0.05). Furthermore, no statistically significant difference was found in the average levels of myelin basic protein between the two subgroups (P>0.05).

## Discussion

In the present study, a clinical investigation was performed to analyze the correlation between the expression of myelin basic protein in the serum and brain metastasis from lung cancer. To the best of our knowledge, few studies have investigated this subject.

Myelin basic protein is specific to the central nerve system and plays an important role in the formation of myelin sheaths and the development of the brain (5). The level of myelin basic protein in the serum may function as a sensitive index for determining the severity, extent and prognosis of nervous system injury (7,12). In the current study, the concentration of myelin basic protein in the serum of the brain metastasis group was significantly higher when compared with the group without brain metastasis. Thus, it was hypothesized that the onset of a metastatic brain tumor damages the blood-brain barrier, causing myelin basic protein to enter the blood, subsequently increasing the levels of myelin basic protein in the serum. To date, the diagnosis of a brain metastasis in lung cancer patients has depended on imaging, particularly CT or MRI (4). However, small brain metastases may be difficult to diagnose early, and in cases where the patients exhibit unstable vital signs, imaging may be difficult to perform. The observations of the present study indicated that the measurement of myelin basic protein may aid physicians in diagnosing the metastasis of lung cancer to the brain.

In addition, no statistically significant difference was found in the serum levels of myelin basic protein between the two subgroups. As shown in Fig. 1, the trend line is flat and the R^2^ value is very low, indicating that the size of the metastatic brain tumor has only a small effect on the serum levels of myelin basic protein. Furthermore, Spearman’s correlation analysis revealed no significant correlation between the size of the metastatic tumor and the serum concentration of myelin basic protein, indicating that even a small metastatic tumor may result in a large increase in the levels of myelin basic protein in the serum. Therefore, brain metastasis may be identified and treated earlier using myelin basic protein as a marker, which may considerably improve the efficacy of treatment and the quality of life of lung cancer patients.

However, there were limitations to the present study. Firstly, the sample size was small, and a larger sample trial may result in a more definite conclusion. In addition, the sensitivity, accuracy and specificity, as well as the cut-off value of myelin basic protein, in diagnosing metastatic brain tumors were not investigated due to the small sample size. These parameters may aid the diagnosis of brain metastasis from lung cancer; thus, further study is required.

Despite the limitations, the results of the present study demonstrated a statistically significant correlation between the expression of myelin basic protein in the serum and the metastasis of lung cancer to the brain. Thus suggesting that myelin basic protein may prove useful in the early diagnosis of brain metastases in patients with lung cancer.

## Figures and Tables

**Table I:** Patient demographics in the two lung cancer groups.

Clinical data	With brain metastasis	Without brain metastasis
Patients, n	32	36
Age, years (range)	54.8 (43–78)	51.6 (39–75)
Gender (male/female), n	21/11	24/12
Lung cancer, n		
Adenocarcinoma	17	16
Small cell carcinoma	10	11
Squamous carcinoma	5	9
Metastasis site, n		
Cerebrum	21	-
Cerebellum	4	-
Cerebrum and cerebellum	3	-
Meninx	2	-
Cerebrum and meninx	2	-

## References

[1] Li Q, Wu H, Chen B (2012). SNPs in the TGF-β signaling pathway are associated with increased risk of brain metastasis in patients with non-small-cell lung cancer. PLoS One.

[2] Han L, Liang XH, Chen LX, Bao SM, Yan ZQ (2013). SIRT1 is highly expressed in brain metastasis tissues of non-small cell lung cancer (NSCLC) and in positive regulation of NSCLC cell migration. Int J Clin Exp Pathol.

[3] Gaspar LE, Scott C, Murray K, Curran W (2000). Validation of the RTOG recursive partitioning analysis (RPA) classification for brain metastases. Int J Radiat Oncol Biol Phys.

[4] Li Y, Li X, Wang Y, Zhou Y (2012). Impact of CT/MRI image registration on target delineation of radiotherapy for lung cancer with brain metastasis. Zhongguo Fei Ai Za Zhi.

[5] Lutz D, Loers G, Kleene R (2014). Myelin basic protein cleaves cell adhesion molecule L1 and promotes neuritogenesis and cell survival. J Biol Chem.

[6] Tzakos AG, Troganis A, Theodorou V (2005). Structure and function of the myelin proteins: current status and perspectives in relation to multiple sclerosis. Curr Med Chem.

[7] Vass K, Lassmann H, Wisniewski HM, Iqbal K (1984). Ultracytochemical distribution of myelin basic protein after injection into the cerebrospinal fluid. Evidence for transport through the blood-brain barrier and binding to the luminal surface of cerebral veins. J Neurol Sci.

[8] D’Aversa TG, Eugenin EA, Lopez L, Berman JW (2013). Myelin basic protein induces inflammatory mediators from primary human endothelial cells and blood-brain barrier disruption: implications for the pathogenesis of multiple sclerosis. Neuropathol Appl Neurobiol.

[9] Gyorgy A, Ling G, Wingo D (2011). Time-dependent changes in serum biomarker levels after blast traumatic brain injury. J Neurotrauma.

[10] Berger RP, Dulani T, Adelson PD, Leventhal JM, Richichi R, Kochanek PM (2006). Identification of inflicted traumatic brain injury in well-appearing infants using serum and cerebrospinal markers: a possible screening tool. Pediatrics.

[11] Baker AJ, Rhind SG, Morrison LJ (2009). Resuscitation with hypertonic saline-dextran reduces serum biomarker levels and correlates with outcome in severe traumatic brain injury patients. J Neurotrauma.

[12] Ide T, Kamijo Y (2008). Myelin basic protein in cerebrospinal fluid: a predictive marker of delayed encephalopathy from carbon monoxide poisoning. Am J Emerg Med.

